# Microbial and Metabolic Dysbiosis in Ruminal Acidosis: Mechanisms, Host Responses, and Microbiota-Targeted Mitigation Strategies

**DOI:** 10.3390/microorganisms14081797

**Published:** 2026-08-14

**Authors:** Yijuan Ma, Xueyong Zhang, Yong Fu, Hong Duo, Cairang Zhouzai

**Affiliations:** 1 Qinghai Provincial Key Laboratory of Pathogen Diagnosis for Animal Diseases and Green Technical Research for Prevention and Control, Academy of Animal Sciences and Veterinary Medicine, Qinghai University, Xining 810016, China; 2 State Key Laboratory for Diagnosis and Treatment of Severe Zoonotic Infectious Diseases, Key Laboratory for Zoonosis Research of the Ministry of Education, Qinghai University, Xining 810016, China; 3 Animal Husbandry and Veterinary Station, Shaliuhe Town, Gangcha County, Xining 810016, China

**Keywords:** ruminal acidosis, microbial dysbiosis, metabolic cross-feeding, ruminal epithelial barrier, host–microbiota interactions, precision nutrition

## Abstract

Ruminal acidosis, particularly subacute ruminal acidosis, remains a major metabolic disorder that compromises animal health, production efficiency, and the sustainability of intensive ruminant systems. Although traditionally defined by reduced ruminal pH, it is increasingly recognized as a multidimensional disorder involving microbial ecological destabilization, disrupted metabolic cross-feeding, impaired epithelial barrier function, and dysregulated host inflammatory responses. This review synthesizes current knowledge of the microbial and metabolic mechanisms underlying acute and subacute ruminal acidosis and highlights processes that extend beyond pH depression alone. High-concentrate feeding shifts the balance among amylolytic and lactate-producing microorganisms, lactate-utilizing populations, and fibrolytic guilds, thereby promoting organic acid accumulation, reducing functional redundancy, and weakening microbial resilience. Concurrent increases in volatile fatty acids, lactate, lipopolysaccharide, histamine, and other microbially derived bioactive compounds increase epithelial acid load, disrupt tight-junction integrity, and facilitate inflammatory signaling. The principal novelty of this review is the integration of microbial functional guilds, metabolic cross-feeding, ecological resilience, epithelial barrier dysfunction, and host inflammation into a unified microbiota–metabolism–barrier–inflammation framework linking dietary perturbation with microbial dysfunction and host pathology. We further critically evaluate nutritional regulation, buffering agents, probiotics, yeast-derived products, postbiotics, and plant bioactive compounds according to their capacity to restore microbial function rather than merely correct ruminal pH. Additionally, this review may support multidimensional risk assessment, guide targeted intervention, and facilitate the integration of continuous ruminal monitoring with precision nutrition for earlier prediction and individualized prevention of ruminal acidosis.

## 1. Introduction

Ruminal acidosis is a common nutritional and metabolic disorder in intensive ruminant production systems and has important consequences for animal health, welfare, and production efficiency. It is generally classified into acute ruminal acidosis (ARA) and subacute ruminal acidosis (SARA) according to the severity and duration of ruminal acidification [1,2]. ARA typically develops after the sudden ingestion of excessive amounts of rapidly fermentable carbohydrates and is characterized by lactate accumulation, severe pH depression, systemic metabolic acidosis, and overt clinical signs. In contrast, SARA involves recurrent or prolonged episodes of moderately depressed ruminal pH, often without obvious symptoms. Because its manifestations are subtle and nonspecific, SARA may remain undetected while impairing feed intake, fiber digestion, milk fat synthesis, and production efficiency [3]. Ruminal pH has traditionally been used as the principal criterion for defining and diagnosing ruminal acidosis. However, pH alone cannot fully represent the biological complexity of this disorder [4,5]. It reflects the balance among acid production, salivary buffering, epithelial absorption, ruminal motility, and digesta passage, and is influenced by diet composition, feeding time, sampling site, and monitoring method. Consequently, animals exposed to similar dietary challenges may show comparable pH values but differ in fermentation profiles, epithelial adaptation, inflammatory responses, and production outcomes. However, how ruminal pH relates to microbial, metabolic, epithelial, and inflammatory changes, and whether these changes are causal or secondary, remains unclear.

The rumen harbors a diverse microbial community composed of bacteria, archaea, protozoa, anaerobic fungi, and viruses. Through trophic interactions and metabolic cross-feeding, these microorganisms convert dietary substrates into volatile fatty acids, microbial protein, and other metabolites essential for host nutrition [6]. Under stable conditions, ruminal homeostasis depends on a coordinated balance among fiber degradation, starch utilization, lactate production and consumption, acid absorption, and buffering. High-concentrate feeding disrupts this balance by favoring amylolytic, acid-tolerant, and lactate-producing microorganisms while suppressing pH-sensitive fibrolytic populations [7,8]. The dominant metabolic features differ between ARA and SARA. In ARA, rapid lactate production may exceed microbial lactate-utilizing capacity, resulting in substantial lactate accumulation and severe ruminal acidification [9,10]. In contrast, persistent lactate accumulation is less common in SARA, in which pH depression is primarily associated with excessive volatile fatty acid production relative to ruminal buffering and epithelial absorptive capacity [11]. Nevertheless, both conditions involve disruption of microbial functional balance. The suppression of fibrolytic populations and an inadequate or delayed response of lactate-utilizing microorganisms may impair fermentation stability, reduce functional redundancy, and weaken the resilience of the ruminal microbial ecosystem [5]. However, it remains unclear whether these microbial shifts precede ruminal acidification or primarily represent secondary and adaptive responses to the altered ruminal environment.

Microbial and metabolic dysbiosis also affects the ruminal epithelium and host physiology [12,13]. Excessive organic acid accumulation increases epithelial acid load, while acidotic conditions promote the release of lipopolysaccharide, histamine, biogenic amines, and other microbial-derived products. Although volatile fatty acids support epithelial development under physiological conditions, excessive concentrations of undissociated acids at low pH may impair tight-junction integrity and increase epithelial permeability. Barrier disruption facilitates the translocation of microbial products and inflammatory stimuli into the portal circulation, linking local fermentation disturbances with hepatic stress, systemic inflammation, impaired production, and secondary disorders.

High-throughput sequencing and multi-omics studies have revealed alterations in microbial composition, functional pathways, metabolite profiles, and epithelial responses during ruminal acidosis [14,15]. However, relative taxonomic abundance does not necessarily reflect microbial activity or metabolic flux, and differences in acidosis models, diagnostic criteria, sampling time points, and analytical methods limit direct comparisons among studies. Accordingly, this review synthesizes current knowledge of microbial and metabolic dysbiosis in ARA and SARA, with particular emphasis on microbial functional guilds, metabolic cross-feeding, ecological stability, epithelial barrier dysfunction, and host inflammatory responses. These processes are integrated into a microbiota–metabolism–barrier–inflammation framework that links dietary perturbation to ruminal dysfunction and production losses. We also critically evaluate the evidence and limitations of microbiota-targeted mitigation strategies and discuss future priorities in biomarker development, multi-omics integration, individual susceptibility assessment, and precision nutrition.

## 2. Definition and Diagnostic Framework of Ruminal Acidosis

### 2.1. Acute and Subacute Ruminal Acidosis

Ruminal acidosis comprises a spectrum of fermentation disorders that differ in their onset, severity, duration, metabolic characteristics, and clinical consequences. It is generally classified into acute ruminal acidosis (ARA) and subacute ruminal acidosis (SARA), although the boundary between these conditions is not always clearly defined [10,16]. ARA is a severe and potentially life-threatening condition that usually develops after the sudden ingestion of large amounts of rapidly fermentable carbohydrates without adequate dietary adaptation. The rapid fermentation of starch and soluble sugars can cause substantial organic acid accumulation, a marked increase in ruminal osmolality, and a rapid decline in ruminal pH, frequently to values below 5.0 [9]. Affected animals may exhibit anorexia, depression, dehydration, diarrhea, reduced ruminal motility, ataxia, recumbency, and, in severe cases, shock or death. The pronounced clinical manifestations and rapid disease progression generally make ARA easier to recognize than SARA.

SARA is more common in intensive dairy and beef production systems and is characterized by recurrent or prolonged periods of moderately depressed ruminal pH without the severe clinical signs observed in ARA [17]. It is typically associated with high-concentrate feeding, insufficient physically effective fiber, abrupt dietary transitions, irregular feed intake, or inadequate adaptation to rapidly fermentable diets [18]. Clinical signs are often subtle and nonspecific and may include reduced or variable dry matter intake, decreased rumination, loose feces, milk fat depression, impaired feed efficiency, and reduced production performance [19]. ARA and SARA also differ in their predominant metabolic characteristics: ARA is commonly associated with substantial lactate accumulation, whereas persistent lactate accumulation in SARA is typically less, in which the decline in ruminal pH is more frequently related to excessive volatile fatty acid production relative to ruminal buffering and epithelial absorptive capacity [20]. Nevertheless, these distinctions should not be interpreted as absolute; the relative contributions of lactate, volatile fatty acids, buffering, absorption, and digesta passage depend on the composition of the diet, the rate of carbohydrate ingestion, previous dietary adaptation, and individual animal susceptibility. Beyond these clinical and metabolic differences, susceptibility to ruminal acidosis also varies across production stages, particularly during periods of dietary transition.

### 2.2. Production Stage Susceptibility and Critical Dietary Transitions

Susceptibility to ruminal acidosis varies across the dairy production cycle because diet composition, feed intake, metabolic demand, and ruminal adaptive capacity change with production stage [18,21]. Transitions between stages are particularly important, as rapid changes in substrate supply may disturb the balance among microbial fermentation, salivary buffering, and epithelial acid absorption [22]. At dry-off, cows are generally shifted from an energy-dense lactation diet to a more forage-based dry-cow ration, resulting in substantial changes in ruminal substrate availability and fermentation. During the close-up period, concentrates are commonly introduced progressively to facilitate adaptation to the postpartum diet. The effectiveness of this adaptation depends on the rate and magnitude of dietary change, as well as on individual feeding behavior and ruminal responsiveness [23].

The transition from late gestation to early lactation represents a particularly important period for SARA development [24]. After calving, the energy requirements for milk production increase rapidly, and diets commonly contain greater proportions of concentrates and rapidly fermentable carbohydrates. However, dry matter intake and feeding patterns may remain unstable, while the ruminal microbial community and epithelium require time to adapt to the increased fermentative acid load [21]. Consequently, abrupt increases in starch intake, inadequate physically effective fiber, ration sorting, large meals, and irregular feed access may increase postprandial acid production and prolong low-pH exposure. Fresh and early-lactation cows may therefore be more susceptible to SARA, although the magnitude and duration of ruminal pH depression vary considerably among individuals. Production stage alone, however, does not fully explain susceptibility to ruminal acidosis. Parity, days relative to calving, previous dietary adaptation, meal patterns, ruminal absorptive capacity, microbial functional resilience, and herd-level feeding management may modify the response to the same dietary challenge. Therefore, studies investigating the etiology and diagnosis of ruminal acidosis should stratify cows according to production stage and report parity, days relative to calving, diet composition, and the rate of dietary transition. Particular attention should be given to the periods surrounding dry-off, prepartum dietary adaptation, calving, and early lactation, because these transitions may influence fermentation stability and individual susceptibility.

### 2.3. Ruminal pH as a Diagnostic Indicator

Ruminal pH remains the most widely used indicator of ruminal acidosis because it reflects the balance among organic acid production, salivary buffering, epithelial absorption, and digesta passage [1]. However, no universally accepted pH threshold or duration criterion has been established for SARA. Values between approximately 5.5 and 5.8 have commonly been used, but the selected threshold and required duration of pH depression vary considerably among studies.

Variation in sampling procedures is an important source of inconsistency. Oral stomach tube samples may be contaminated with saliva and generally yield higher pH values than samples collected through a ruminal cannula, whereas rumenocentesis provides more localized measurements but is invasive and unsuitable for repeated monitoring. Indwelling sensors can continuously characterize postprandial pH decline and recovery, although sensor position, calibration drift, and differences between ruminal and reticular measurements may influence the results. Villot et al. [17] showed that indicators incorporating the duration and magnitude of pH depression can improve SARA detection compared with isolated pH measurements. Accordingly, the time below a selected threshold, area under the threshold, rate of postprandial decline, and recovery time may better represent acidotic burden than a single minimum value. However, their interpretation remains affected by the choice of threshold, monitoring device, measurement site, and experimental model. In addition, variation in breed, production type, physiological stage, and individual animal characteristics limits the application of uniform pH thresholds. Dairy and beef cattle differ in feeding systems and production demands, while dry, transition, fresh, and lactating cows differ in diet composition, feed intake, and ruminal adaptation. Even within the same production group, animals exposed to similar diets may show different magnitudes and durations of pH depression because of variation in feeding behavior, saliva production, ruminal volume, microbial functional capacity, and epithelial absorption [25,26]. Therefore, ruminal pH should be interpreted in relation to animal type, production stage, diet, and monitoring method.

### 2.4. Limitations of a pH Centered Definition

Although ruminal pH remains the most widely used indicator for defining ruminal acidosis, pH-based definitions have important limitations. Similar pH profiles may arise from different combinations of VFA production, lactate accumulation, salivary buffering, epithelial transport, ruminal motility, water movement, and digesta passage. Penner et al. [20] emphasized that epithelial adaptation and short-chain fatty acid absorption are important determinants of ruminal pH regulation and susceptibility to SARA. In addition, individual variation further limits the application of uniform pH thresholds. Animals exposed to similar high-concentrate diets frequently differ in the magnitude and duration of pH depression, indicating that feeding behavior, saliva production, ruminal volume, previous dietary adaptation, microbial functional capacity, and host epithelial responses all influence susceptibility [25]. Petri et al. [26] also demonstrated that dietary adaptation and induced acidosis alter the rumen epimural bacterial community, suggesting that microbial and epithelial characteristics contribute to inter-animal differences. A transient decline in ruminal pH following feed intake should be distinguished from pathological acidosis, as a moderate decrease may represent a normal adaptive response when VFA production is balanced by salivary buffering, epithelial absorption, and microbial utilization. Volatile fatty acids are major energy substrates for ruminants and, under physiological conditions, contribute to ruminal epithelial development and function [27]. Thus, a moderate decline in pH may reflect a normal postprandial response when acid production is adequately counterbalanced by salivary buffering, epithelial absorption, and microbial utilization. In contrast, pathological acidosis is more likely to occur when the rate, magnitude, or duration of acid accumulation exceeds the buffering and adaptive capacity of the ruminal microbial ecosystem and epithelium.

A multidimensional diagnostic framework should therefore combine pH dynamics with fermentation, microbial, epithelial, and host indicators. Total and individual VFA concentrations, lactate, ruminal osmolality, and the balance among acid-producing, lactate-utilizing, and fibrolytic microbial groups may provide information on fermentation stability [28]. Markers of epithelial integrity, microbial product translocation, and systemic inflammation, together with alterations in feeding behavior and production performance, may provide complementary information for assessing the biological consequences and severity of ruminal acidosis [29]. Future studies should therefore distinguish early predictive biomarkers from downstream pathological markers and validate integrated diagnostic models under commercial production conditions. Overall, ruminal acidosis is better regarded as a continuum of microbial, metabolic, epithelial, and host disturbances than as a disorder defined by a single pH threshold. Ruminal pH remains a central diagnostic variable, but it should be interpreted together with the duration and magnitude of acidification, the underlying fermentation profile, and the adaptive responses of the microbiota and host.

## 3. Microbial Ecological Dysbiosis During Ruminal Acidosis

The rumen microbiota is a highly integrated ecosystem in which bacteria, protozoa, anaerobic fungi, and archaea interact through substrate competition, metabolic cross-feeding, and interspecies hydrogen transfer [30]. Functional redundancy normally allows major fermentation processes to remain stable despite fluctuations in individual microbial populations and contributes to ecosystem resistance and recovery following dietary disturbances [31]. However, microbial responses to an acidotic challenge are shaped not only by dietary composition and ruminal pH but also by production stage, dietary transition, host physiological status, and environmental stress. During the transition from the dry period to early lactation, marked changes in diet composition, dry matter intake, metabolic demand, and ruminal adaptation can reshape the ruminal microbial community [32,33]. Therefore, microbiota changes observed around calving should be interpreted in the context of concurrent dietary and physiological changes rather than being attributed to ruminal acidosis alone.

Parturition-associated metabolic and oxidative stress may further affect immune and epithelial homeostasis during the transition period [34]. Heat stress can also reduce dry matter intake and rumination, alter ruminal motility and fermentation, and modify the ruminal microbial community. These stressors are therefore more appropriately regarded as factors that modify susceptibility to diet-induced microbial dysbiosis rather than as independent causes of ruminal acidosis [35]. High-concentrate feeding disrupts this ecological balance by rapidly increasing the availability of starch and soluble carbohydrates while reducing the relative contribution of structural carbohydrates. Integrated microbiome–metabolome analyses have shown that increasing dietary grain shifts ruminal fermentation from fiber-associated metabolism toward the rapid utilization of non-structural carbohydrates [6]. However, Khafipour et al. [36] observed distinct bacterial community responses between grain and alfalfa pellet-induced SARA models despite both producing ruminal acidification. These findings indicate that microbial dysbiosis is influenced not only by pH depression but also by substrate type, fermentation pattern, and challenge duration. Ruminal acidosis should therefore be regarded as a functional reorganization of the microbial ecosystem rather than a uniform alteration in a limited number of microbial taxa.

### 3.1. Functional Reorganization of Bacterial Guilds

The bacterial response to ruminal acidosis is characterized by an altered balance among acid-producing, acid-utilizing, fibrolytic, and metabolically versatile functional guilds. Rapidly fermentable carbohydrate overload is commonly associated with increased representation or activity of starch-degrading and lactate-producing bacteria, including *Streptococcus bovis* and acid-tolerant Lactobacillus species [9,10,11,37]. Russell and Hino [11] demonstrated that enhanced lactate production by *S. bovis* can initiate a self-reinforcing process in which acid production accelerates the decline in ruminal pH. Nevertheless, lactate accumulation depends on the balance between production and utilization. *Megasphaera elsdenii* and some strains of *Selenomonas ruminantium* convert lactate into propionate and other fermentation products, but their growth and metabolic responses may lag behind rapid lactate production during sudden carbohydrate overload [38,39]. Chen et al. [40] further showed that the lactate-degrading pattern of *M. elsdenii* varies according to pH, lactate concentration, and acidosis model. ARA may therefore result from a temporal mismatch between lactate production and community-level lactate clearance rather than from the complete absence of lactate-utilizing bacteria.

At the same time, low ruminal pH suppresses major fibrolytic bacteria, including *Fibrobacter succinogenes, Ruminococcus albus, and Ruminococcus flavefaciens* [41,42]. Reduced fibrolytic activity is associated with impaired fiber degradation and may weaken particle-associated microbial interactions and fermentation stability, and it may indirectly reduce rumination and salivary buffering as well. Li et al. [41] linked the reduced representation of fiber-associated populations with a greater risk of SARA and altered fermentation in lambs. In contrast, metabolically versatile taxa such as Prevotella show inconsistent responses because species within this genus differ markedly in their ability to utilize starch, proteins, peptides, and hemicellulose [6,36,38]. Genus-level abundance therefore cannot reliably indicate whether these organisms contribute to acid production, protein metabolism, fiber degradation, or adaptive substrate utilization. Overall, bacterial dysbiosis reflects a shift from a functionally redundant, fiber-supporting community toward a more acidogenic and less stable microbial state.

### 3.2. Disruption of Microbial Cross-Feeding

Ruminal fermentation depends on the sequential transfer of intermediate metabolites among microbial populations. Lactate, succinate, hydrogen, formate, and soluble oligosaccharides produced by primary degraders are subsequently utilized by secondary fermenters, thereby preventing excessive accumulation of intermediates and improving overall fermentation efficiency [38]. Lactate cross-feeding is particularly important during abrupt adaptation to high-concentrate diets and under conditions in which excessive lactate production may predispose animals to ARA. Lactate produced by rapidly growing amylolytic bacteria is normally consumed by *M. elsdenii* and lactate-utilizing strains of *S. ruminantium* [39]. However, the efficiency of this interaction depends on the abundance, metabolic activity, and response time of both producing and utilizing populations. The environment-dependent lactate utilization observed by Chen et al. [40] indicates that the presence of *M. elsdenii* alone does not guarantee sufficient lactate clearance.

Similar interactions occur in succinate and hydrogen metabolism, succinate produced by several starch- and hemicellulose-degrading bacteria can be converted to propionate by other community members [43], whereas hydrogen generated by bacteria, protozoa, and fungi is consumed by methanogenic archaea. Disruption of these pathways may alter redox balance and redirect fermentation toward alternative end products even when total microbial abundance remains relatively stable [44]. These observations suggest that ruminal dysfunction may arise from the loss of metabolic coordination rather than the disappearance of a specific organism. Nevertheless, most current evidence for disrupted cross-feeding is based on correlations between microbial abundance and metabolite concentration [45]. Stable isotope tracing, coculture experiments, metatranscriptomics, and metabolic flux analysis are needed to demonstrate the direction and rate of metabolite transfer during the onset and recovery of ruminal acidosis [45].

### 3.3. Responses of Protozoa, Anaerobic Fungi, and Archaea

Protozoa, anaerobic fungi, and archaea also contribute to ruminal ecosystem stability, despite bacteria receiving the greatest attention, with protozoa playing important roles in starch sequestration, bacterial predation, and hydrogen metabolism [7]. Belanche et al. [46] demonstrated that variation in protozoal community structure can markedly affect ruminal fermentation, bacterial diversity, and methane production. Many protozoal taxa are sensitive to sustained reductions in ruminal pH, and their decline may decrease starch sequestration, thereby increasing the availability of rapidly fermentable substrates to amylolytic bacteria [47]. However, protozoa can also contribute to starch fermentation, lactate formation, and hydrogen production [48]. Their effects during ruminal acidosis are therefore context-dependent and cannot be regarded as uniformly beneficial or detrimental. Anaerobic fungi are primary colonizers of fibrous plant material and promote lignocellulose degradation through mechanical penetration of plant tissues and the production of fibrolytic enzymes [49]. Acidic conditions may impair fungal growth, zoospore activity, and colonization of feed particles, thereby compromising the initial breakdown of structural carbohydrates and reducing substrate availability for downstream fibrolytic populations [50]. Methanogenic archaea may also be affected indirectly because their metabolism depends largely on hydrogen and formate supplied by bacteria, protozoa, and anaerobic fungi [48]. High-concentrate feeding is frequently associated with reduced methane production, although this response may result from several mechanisms, including inhibition associated with low ruminal pH, reduced hydrogen availability, and redirection of reducing equivalents toward propionate forming pathways.

Together, these observations suggest that ruminal acidosis represents a cross-domain ecological disturbance rather than a bacterial disorder alone. Nevertheless, the responses of protozoa, anaerobic fungi, and archaea remain incompletely understood, partly because estimates of their abundance and diversity are influenced by DNA extraction procedures, primer selection, reference-database coverage, and taxonomic resolution [51].

### 3.4. Network Instability and Ecological Resilience

Microbial ecosystem stability depends not only on taxonomic diversity but also on the organization of ecological interactions. Functional redundancy allows multiple microorganisms to perform similar metabolic functions and reduces the impact of fluctuations in individual populations [31]. Mizrahi and Jami [52] suggested that substantial inter-animal variation in rumen microbial community composition can coexist with relatively conserved fermentation functions, reflecting the functional redundancy and resilience of the rumen ecosystem. During high-concentrate feeding, the suppression of pH-sensitive organisms and increasing dependence on a narrower range of acid-tolerant populations may reduce this ecological buffering capacity. Network-based studies provide additional evidence that acidosis affects microbial organization rather than only individual taxa. Mu et al. [13] observed coordinated changes in microbial communities, metabolites, and epithelial responses during high grain-induced SARA. Guo et al. [14] further reported that yeast-derived postbiotics modified microbial network interactions and increased the diversity of hub taxa during grain-based SARA challenges. These findings suggest that the maintenance or restoration of microbial connectivity may be an important mechanism underlying ruminal stability. However, co-occurrence networks are based mainly on statistical correlations and do not necessarily represent direct biological interactions. Network structure is also sensitive to sample size, data transformation, taxonomic resolution, and the thresholds used to define associations.

Differences in ecological resilience may partly explain individual susceptibility to ruminal acidosis. Animals receiving similar diets can differ markedly in pH dynamics, fermentation patterns, microbial composition, and production responses [20,26]. A resilient microbial community may rapidly increase acid-utilizing activity, preserve fibrolytic function, and recover after a dietary challenge, whereas a susceptible community may show delayed adaptation and prolonged loss of key metabolic interactions. Most current studies use one or a few sampling time points and therefore cannot distinguish resistance to the initial disturbance from recovery after the disturbance. Longitudinal studies covering baseline, challenge, and recovery periods are needed to quantify microbial resistance, recovery rate, and functional stability [53]. Collectively, ruminal acidosis involves a loss of coordination among microbial functional guilds rather than a simple replacement of beneficial microorganisms by harmful taxa. Rapid carbohydrate fermentation, suppression of fibrolytic activity, disruption of metabolic cross-feeding, and reduced ecological resilience collectively drive microbial dysbiosis, while delayed lactate clearance is particularly relevant to ARA and abrupt carbohydrate overload. Future studies should therefore move beyond descriptive taxonomic comparisons and integrate absolute microbial abundance, functional activity, temporal dynamics, and experimentally validated interactions. The major microbial functional groups and ecological alterations associated with ruminal acidosis are summarized in Table 1.

## 4. Metabolic Consequences of Microbial–Derived Dysbiosis

Metabolic dysbiosis represents the functional consequence of microbial ecological disruption during ruminal acidosis [5]. High-concentrate feeding alters the production, utilization, and removal of fermentation products, leading to excessive organic acid accumulation and changes in bioactive compounds [9,16]. These alterations are not determined solely by the abundance of individual microorganisms but also by substrate availability, metabolic flux, epithelial absorption, and the temporal coordination of microbial cross-feeding [56]. Integrated microbiome–metabolome studies have shown that increasing dietary grain produces coordinated changes in microbial functions and ruminal metabolite profiles [13,57]. Ruminal acidosis should therefore be interpreted as a disturbance of metabolic balance rather than simply an increase in total acid production.

### 4.1. Organic Acid Imbalance and Altered Fermentation Profiles

VFAs, principally acetate, propionate, and butyrate, are the major end products of ruminal carbohydrate fermentation and provide a substantial proportion of the host’s metabolizable energy. Under normal conditions, their production is balanced by ruminal buffering, epithelial absorption, and digesta passage. High-concentrate feeding accelerates starch and sugar fermentation, and acid production may exceed the capacity for absorption and neutralization, resulting in a progressive decline in ruminal pH [5,58]. In SARA, this imbalance is generally associated with increased total VFA production rather than sustained lactate accumulation [1,16]. The fermentation profile also commonly shifts toward greater propionate production and a lower acetate-to-propionate ratio, reflecting reduced fibrolytic activity and increased non-structural carbohydrate utilization [6,36]. The relationship between VFA concentration and acidosis is nevertheless not straightforward. A high VFA concentration may indicate rapid fermentation, limited epithelial absorption, reduced ruminal fluid volume, or a combination of these factors. Furthermore, acetate, propionate, and butyrate have distinct metabolic and epithelial effects, and changes in molar proportion do not necessarily correspond to changes in absolute concentration. Therefore, total VFA concentration alone cannot distinguish adaptive high-rate fermentation from pathological acid accumulation.

Lactate plays a more prominent role in ARA. Under stable conditions, lactate remains at a low concentration because it is rapidly utilized by secondary fermenters. Following abrupt carbohydrate overload, however, lactate production may exceed microbial lactate clearance, leading to rapid and severe acidification [9,10]. Chen et al. [40] showed that lactate degradation by *Megasphaera elsdenii* varies with environmental pH, lactate concentration, and acidosis model, supporting the view that lactate accumulation reflects both increased production and insufficient utilization. Collectively, ARA and SARA differ in their dominant organic acid profiles but share a common imbalance between acid production and removal. The metabolic outcome depends on the rate of substrate fermentation, microbial cross-feeding, salivary buffering, epithelial transport, ruminal volume, and passage rate. Most studies measure metabolite concentrations at a limited number of time points and therefore cannot directly determine production and clearance rates. Longitudinal sampling, isotope tracing, and metabolic flux analysis are required to distinguish increased acid production from impaired utilization or absorption.

### 4.2. Lipopolysaccharide and Other Microbial Structural Products

In addition to fermentation acids, ruminal acidosis is associated with increased exposure to microbial structural products. LPS is a component of the outer membrane of Gram-negative bacteria and should be distinguished from microbial metabolites. Acid stress, changes in bacterial community structure, and bacterial lysis may increase the amount of free LPS in ruminal fluid [56]. Gozho et al. [59] reported that grain-induced SARA increased ruminal LPS concentrations and was accompanied by inflammatory responses. Khafipour et al. [60] subsequently showed that a grain-based SARA challenge was associated with evidence of LPS translocation and systemic inflammatory activation. These findings support a potential role for LPS in linking ruminal microbial disruption with host inflammatory response [61]. Furthermore, increased ruminal LPS does not necessarily result in proportional systemic exposure, because translocation is additionally influenced by epithelial permeability, local detoxification, portal circulation, and hepatic clearance [62,63]. Other microbial-associated molecular patterns, including peptidoglycan fragments, lipoteichoic acid, bacterial DNA, and extracellular vesicles, may also contribute to epithelial and immune activation. However, these components have received substantially less attention than LPS in studies of ruminal acidosis. The current evidence base is therefore strongly centered on LPS and may underestimate the diversity of microbial structural signals involved in host responses.

### 4.3. Histamine and Other Biogenic Amines

Biogenic amines are formed primarily through the microbial decarboxylation of amino acids. Histamine, produced from histidine, is among the most extensively studied amines in ruminal acidosis. High-concentrate feeding, low pH, and increased availability of amino acid substrates may favor histamine accumulation [62,64]. Gao et al. [64] demonstrated that histamine and LPS directly increased ruminal epithelial permeability in an ex vivo model, whereas Sun et al. [65] showed that histamine induced inflammatory responses in bovine ruminal epithelial cells through NF-κB-related signaling. These findings indicate that histamine is not merely a marker of abnormal fermentation but a biologically active compound capable of affecting epithelial and inflammatory processes. However, ruminal histamine concentrations vary substantially among studies, and its accumulation depends on dietary histidine supply, microbial decarboxylase activity, degradation pathways, sampling time, and ruminal pH. Other biogenic amines, including tyramine, putrescine, and cadaverine, may also increase during microbial dysbiosis [66]. These compounds can influence epithelial function, vascular responses, oxidative stress, and immune regulation, but their roles in ruminal acidosis remain less clearly established. Most studies report concentrations without identifying the microbial producers, metabolic pathways, or exposure thresholds associated with host injury.

### 4.4. Emerging Metabolic Pathways and Metabolomic Signatures

Metabolomic studies have expanded the understanding of ruminal acidosis beyond VFAs, lactate, LPS, and biogenic amines. High-grain feeding can alter amino acid metabolism, lipid-related pathways, oxidative stress associated metabolites, and microbial host signaling molecules [6,13]. These broader metabolic changes may help explain why animals with similar pH profiles exhibit different epithelial and systemic responses. Tryptophan metabolism has emerged as a potentially important pathway. Chen et al. [67] linked alterations in microbial tryptophan metabolism with ruminal epithelial inflammation during SARA. Microbial indole derivatives may regulate epithelial barrier function and immune homeostasis through several host signaling pathways [68]. These observations suggest that the biological consequences of ruminal dysbiosis may depend not only on toxic or inflammatory compounds but also on the loss of metabolites that normally support epithelial homeostasis. Metabolomic signatures have potential value for identifying early or subclinical disturbances, particularly when combined with microbial, epithelial, and physiological data. Nevertheless, metabolomic findings are often inconsistent across studies because of differences in animal species, diet composition, sampling matrix, sample preparation, analytical platform, and metabolite annotation. Overall, metabolic dysbiosis in ruminal acidosis involves altered organic acid flux, increased exposure to microbial structural products, accumulation of bioactive amines, and broader changes in microbial–host signaling metabolite. Table 2 summarizes the major fermentation metabolites and microbial-derived bioactive products associated with ruminal acidosis.

## 5. Host Responses to Ruminal Acidosis

The host response to ruminal acidosis is initiated when microbial and metabolic perturbations exceed the adaptive capacity of the ruminal epithelium. As the principal interface between the rumen microbiota and the host, the ruminal epithelium serves the dual functions of absorbing fermentation products and limiting the translocation of luminal microorganisms and their bioactive components into host tissue [72]. During prolonged or severe acidotic challenge, excessive exposure to organic acids and microbial-derived bioactive compounds, together with disturbances in epithelial metabolism, may impair both absorptive and barrier functions [52,73]. Subsequent consequences include disruption of epithelial integrity, activation of local inflammatory pathways, increased delivery of rumen-derived products to the liver, impaired nutrient utilization, and the development of secondary health disorders [73,74,75].

### 5.1. Ruminal Epithelial Barrier Dysfunction

The ruminal epithelium possesses considerable capacity to adapt to increased fermentation and VFA supply. At physiological concentrations, VFAs, particularly butyrate, provide energy for epithelial cells and promote epithelial growth, differentiation, and absorptive development [56]. However, when organic acid production exceeds epithelial transport and intracellular buffering capacity, the increased concentration of undissociated acids can enhance proton entry into epithelial cells, disturb intracellular pH homeostasis, and impair membrane function. Meissner et al. [74] demonstrated that short-chain fatty acids contributed directly to epithelial barrier failure under acidotic conditions, indicating that epithelial injury depends on the combined effects of acid concentration and extracellular pH rather than pH depression alone.

Barrier integrity also depends on intercellular tight-junction complexes, including claudins, occludin, and zonula occludens proteins. Prolonged exposure to low-pH and microbial-derived products can alter epithelial morphology, tight-junction expression, and paracellular permeability [73,75]. Emmanuel et al. [73] showed that acidic conditions and LPS increased the permeability of ruminal and colonic tissues, whereas Gao et al. [64] reported that both LPS and histamine directly impaired ruminal epithelial permeability in an ex vivo model. Collectively, these findings suggest that barrier dysfunction results from the interaction between acid stress and microbial bioactive products rather than from a single injurious factor. Nevertheless, changes in tight-junction gene or protein expression do not necessarily correspond to functional barrier failure. In vitro and ex vivo systems are useful for identifying direct mechanisms but cannot fully reproduce epithelial adaptation, blood flow, immune-cell recruitment, and metabolite clearance in vivo.

### 5.2. Activation of Local Inflammatory Signaling

The ruminal epithelium is not only a physical barrier but also an active component of mucosal immunity. When epithelial integrity is impaired, LPS and other microbial-associated molecular patterns can interact with pattern-recognition receptors expressed by epithelial and immune cells [72]. LPS recognition involves LPS-binding protein, CD14, MD2, and TLR4 [71], followed by activation of downstream NF-κB and MAPK signaling pathways and increased expression of inflammatory mediators, including TNF-α, IL-1β, and IL-6 [73]. Kent-Dennis et al. [75] detected localized immune responses in ruminal epithelial tissue following a short-term SARA challenge, supporting the capacity of the ruminal mucosa to respond directly to acidotic disturbances. Sun et al. [65] showed that histamine activated NF-κB-related inflammatory signaling in bovine ruminal epithelial cells. These findings support a feed-forward mechanism in which acid stress and microbial products damage the epithelial barrier, increased permeability enhances exposure to inflammatory stimuli, and inflammation further compromises epithelial integrity. However, inflammatory responses are not consistent across all SARA models. Grain-induced challenges frequently increase ruminal LPS release and inflammatory markers, whereas other dietary models producing similar pH depression may induce weaker or variable responses.

### 5.3. Hepatic Exposure and the Rumen-Liver Connection

Microbial products that cross the ruminal epithelium enter the portal circulation and are transported first to the liver. Under physiological conditions, hepatic Kupffer cells and other innate immune mechanisms contribute to the recognition, detoxification, and clearance of these compounds. During ruminal acidosis, increased epithelial permeability may enhance hepatic exposure to LPS and other microbial-derived products, thereby increasing both inflammatory and metabolic demands on the liver [65]. Repeated or excessive exposure to these compounds can increase hepatic immune activation and metabolic burden. Activation of hepatic inflammatory pathways may promote the production of acute-phase proteins and pro-inflammatory mediators, while also interfering with nutrient metabolism [76]. Repeated exposure to microbial products may activate hepatic NF-κB and related pathways, increase acute-phase responses, impair antioxidant capacity, and interfere with glucose and lipid metabolism [77]. This hepatic burden may be particularly important in high-producing dairy cows because the liver already has substantial demands for gluconeogenesis, fatty acid oxidation, and the metabolic provision of precursors required for milk synthesis [78]. Inflammatory activation can redirect nutrients toward immune defense and tissue repair, thereby reducing the resources available for production [79]. Nevertheless, the detection of LPS or inflammatory markers in peripheral blood does not establish the rumen as their exclusive source, because endotoxins may also originate from the hindgut, mammary gland, uterus, or other infected and barrier-compromised tissues [80].

### 5.4. Effects on Feed Intake and Production Performance

The host response to ruminal acidosis is ultimately reflected in changes in feeding behavior and production. SARA is frequently associated with reduced or variable dry matter intake, decreased rumination, altered meal patterns, and impaired feed efficiency [16]. Keunen et al. [19] found that experimentally induced SARA altered diet selection in dairy cows, suggesting that changes in feeding behavior may represent an adaptive response to ruminal discomfort and metabolic disturbance. Increased ruminal osmolality, reduced motility, epithelial inflammation, and circulating inflammatory mediators may all contribute to appetite suppression.

The effects of SARA on milk yield and composition vary according to the induction model, diet composition, challenge duration, lactation stage, and individual susceptibility. Krause and Oetzel [81] reported that milk yield decreased from 35.2 to 31.7 kg/d during experimentally induced grain-based SARA, corresponding to a reduction of approximately 10%. Khafipour et al. [82] found that alfalfa pellet-induced SARA reduced milk yield from 35.9 to 32.7 kg/d, milk fat concentration from 3.22% to 2.32%, and milk fat yield from 1.14 to 0.77 kg/d. In addition, Ventto et al. [69] demonstrated that diet-induced milk fat depression was associated with altered ruminal biohydrogenation pathways and the formation of specific fatty acid intermediates that suppress mammary lipid synthesis. However, changes in other milk-quality indicators, including lactose concentration, milk urea nitrogen, and somatic cell count, have been less consistently reported and may also be influenced by lactation stage, nutrient intake, inflammatory status, and mammary health. Therefore, these indicators should be interpreted together with ruminal pH dynamics and dietary conditions rather than being regarded as specific markers of SARA. Milk fat depression is another important production consequence. Although a lower acetate-to-propionate ratio may reduce the availability of lipogenic substrates, changes in ruminal biohydrogenation are also central to this response. Ventto et al. [69] demonstrated that diet-induced milk fat depression was associated with altered biohydrogenation pathways and the formation of specific fatty acid intermediates capable of suppressing mammary lipid synthesis. Ruminal acidosis therefore affects milk composition through both altered nutrient precursor supply and the generation of bioactive fermentation products [83]. In addition, during systemic immune activation, nutrients may be redirected from productive processes toward immune defense and tissue repair. Consequently, even subclinical rumen acidosis can reduce production efficiency and increase economic losses in dairy systems.

### 5.5. Secondary Health Disorders

**Figure 1:**
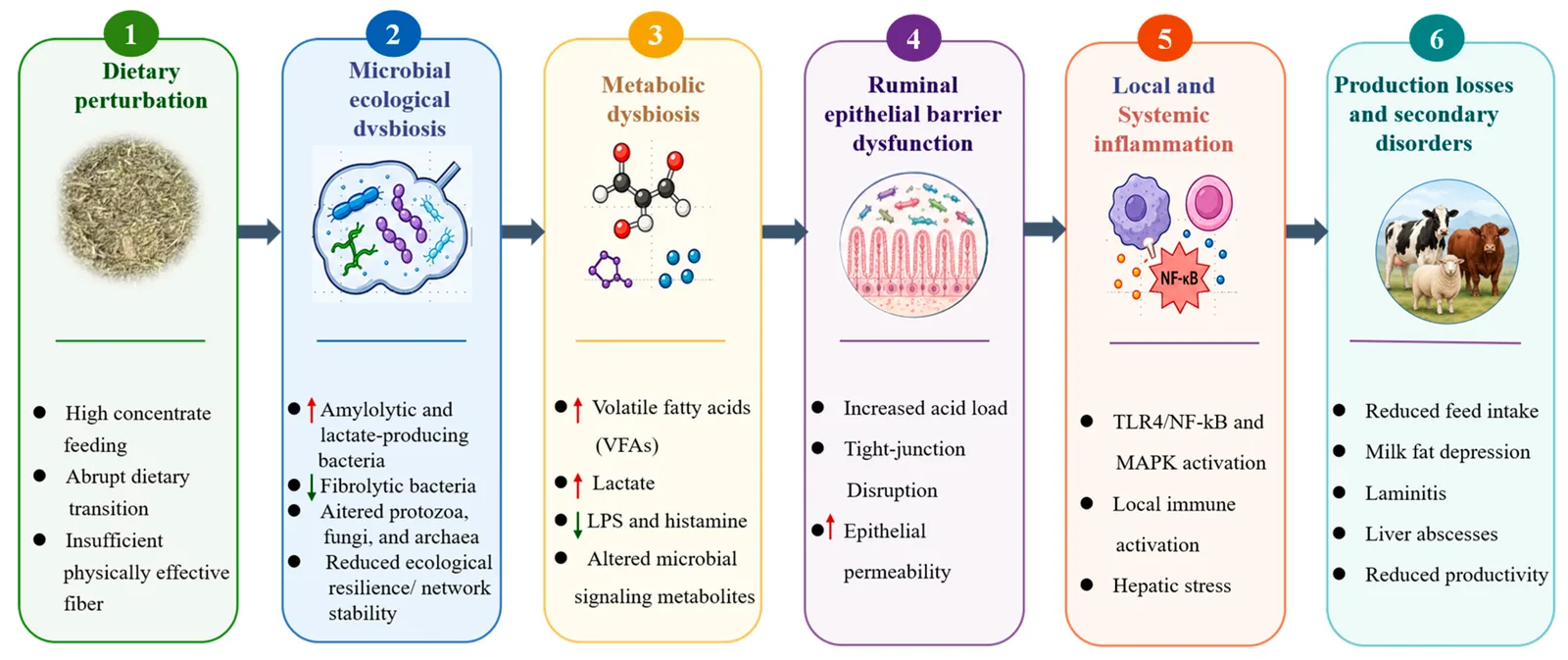
The microbiota–metabolism–barrier–inflammation cascade in ruminal acidosis. Dietary perturbations induce ruminal microbial dysbiosis, characterized by increased amylolytic and lactate-producing bacteria, reduced fibrolytic populations, altered protozoal, fungal, and archaeal communities, and decreased ecological resilience. The resulting increases in volatile fatty acids, lactate, lipopolysaccharide, histamine, and other microbial signaling metabolites increase epithelial acid load, disrupt tight junctions, and enhance ruminal permeability. Activation of TLR4/NF-κB- and MAPK-related pathways then promotes local inflammation and hepatic stress, contributing to reduced feed intake, milk fat depression, laminitis, liver abscesses, and impaired productivity. Abbreviations: VFA, volatile fatty acid; LPS, lipopolysaccharide; TLR4, Toll-like receptor 4; NF-κB, nuclear factor kappa B; MAPK, mitogen-activated protein kinase. The arrows between panels indicate the proposed progression of the cascade, while red upward arrows and green downward arrows indicate increases and decreases, respectively.

Repeated ruminal epithelial injury and exposure to microbial-derived products can increase susceptibility to several secondary disorders. Laminitis has frequently been associated with ruminal acidosis, although its pathogenesis remains multifactorial. LPS, histamine, inflammatory cytokines, vascular dysfunction, and impaired microcirculation have all been proposed to contribute to lamellar tissue injury [84]. Nevertheless, housing conditions, claw conformation, mechanical loading, and local tissue responses also influence disease development. The relationship between ruminal acidosis and laminitis should therefore be regarded as biologically plausible but not attributable to a single circulating mediator. Liver abscess formation follows a more clearly defined route. Damage to the ruminal epithelium can allow opportunistic bacteria, particularly Fusobacterium necrophorum and Trueperella pyogenes, to enter the portal circulation and colonize hepatic tissue [85]. This process differs from the translocation of soluble microbial products because it involves viable bacterial invasion following rumenitis. Distinguishing between endotoxin exposure and bacterial infection is therefore important when interpreting hepatic complications. Ruminal acidosis may also affect the lower gastrointestinal tract. When excessive fermentable carbohydrates escape ruminal digestion, increased hindgut fermentation can reduce intestinal pH, alter microbial communities, and impair intestinal barrier function [86]. These changes may contribute to loose feces, diarrhea, and additional exposure to microbial products. Consequently, systemic responses observed during high-concentrate feeding may arise from combined disturbances in the rumen and hindgut rather than from the rumen alone. Overall, host responses to ruminal acidosis progress from epithelial acid stress and barrier dysfunction to local inflammatory activation, hepatic exposure, altered nutrient partitioning, and secondary disease. The major mechanisms linking dietary perturbation to microbial dysbiosis, metabolic disturbance, epithelial barrier dysfunction, inflammation, and production losses are summarized in Figure 1.

## 6. Microbiota-Targeted Mitigation Strategies

Rumen acidosis is not only a nutritional disorder caused by excessive intake of rapidly fermentable carbohydrates, but also a microbial ecological disturbance characterized by altered fermentation patterns, reduced ruminal pH, impaired microbial networks, and increased production of inflammatory metabolites [12]. Therefore, mitigation strategies should aim not only to restore ruminal pH but also to maintain microbial diversity, stabilize fermentation, enhance lactate utilization, and protect rumen epithelial barrier function [87]. Current and emerging approaches include dietary regulation, buffering agents, probiotics- and yeast-derived products, plant bioactive compounds, and precision microbiome modulation.

### 6.1. Dietary Adaptation and Carbohydrate Management

Dietary management remains the primary strategy for preventing ruminal acidosis because it directly controls the amount and rate of fermentable substrate entering the rumen. Abrupt increases in concentrate intake can exceed the adaptive capacity of both the ruminal microbiota and epithelium, whereas gradual dietary transition allows amylolytic, lactate-utilizing, and acid-absorbing functions to adjust more progressively [9,18]. Adequate physically effective neutral detergent fiber promotes chewing, rumination, salivary secretion, and ruminal motility and supports particle-associated fibrolytic communities [88,89]. The source and processing of starch are equally important. Finely ground or highly processed grains generally ferment more rapidly than less extensively processed starch sources, increasing the rate of postprandial acid production [90]. Feeding frequency, ration sorting, meal size, and competition at the feed bunk can further influence daily pH fluctuations even when the formulated forage-to-concentrate ratio remains unchanged. Consequently, dietary risk should be evaluated according to fermentability and actual feeding behavior rather than concentrate proportion alone. Humer et al. [18] emphasized that effective SARA prevention requires coordinated management of dietary adaptation, physically effective fiber, starch degradability, and feed delivery. Collectively, these measures reduce the initial acidogenic pressure and create conditions that favor microbial adaptation.

Dietary management should also account for production stage. During the dry and close-up periods, gradual adaptation to the more fermentable diets used after calving is important because ruminal epithelial transport and absorptive capacity require time to adjust to changes in short-chain fatty acid supply [22]. After calving, particularly during early lactation, increasing nutrient requirements often necessitate greater concentrate supplementation, which can substantially alter ruminal fermentation and reticular pH. Falk et al. [91] showed that concentrate supplementation during early lactation affected ruminal fermentation and reticular pH and that responses differed between cows with different milk-production potentials. Production stage itself may also influence susceptibility to acidotic challenge. Dohme et al. [92] reported that early-lactation cows fed a lower-forage diet had lower mean and nadir ruminal pH and experienced longer periods below SARA thresholds than mid-lactation cows receiving a higher-forage diet. These findings indicate that dietary strategies for preventing SARA should consider production stage together with forage supply, concentrate intake, dietary adaptation, and individual production demand rather than applying a uniform feeding strategy throughout lactation. However, increasing fiber or reducing starch excessively may lower dietary energy density and compromise production. Dietary management must therefore balance fermentation stability with the nutrient requirements of high-producing animals.

### 6.2. Buffers and Alkalizing Agents

Buffers and alkalizing agents are commonly used to reduce the magnitude and duration of ruminal pH depression. Sodium bicarbonate increases ruminal buffering capacity by neutralizing hydrogen ions, whereas magnesium oxide has a stronger alkalizing effect and may complement bicarbonate-based supplementation [93]. By moderating low-pH exposure, these agents may indirectly preserve fibrolytic activity, support microbial cross-feeding, and reduce epithelial acid stress. Marden et al. [55] compared live yeast with sodium bicarbonate in high-producing dairy cows and showed that both approaches could stabilize ruminal pH through partly distinct mechanisms. Buffering agents primarily act through physicochemical neutralization, whereas yeast products may additionally influence microbial activity. More recent studies have suggested that carbonate-based mixtures can improve pH stability and modify fermentation or microbial profiles [94]. Nevertheless, microbial changes observed after buffer supplementation are generally secondary to modification of the ruminal environment rather than direct microbiota targeting. The efficacy of buffers depends on basal diet, dose, intake consistency, feeding frequency, and the severity of the acidotic challenge [95]. An improvement in mean ruminal pH does not necessarily indicate restoration of metabolic cross-feeding or epithelial barrier function. Excessive or poorly balanced mineral supplementation may also alter dietary cation–anion balance or increase feeding costs.

### 6.3. Probiotics, Direct-Fed Microbials, Yeast, and Postbiotics

Bacterial probiotics and direct-fed microbials can modulate ruminal fermentation by introducing microorganisms with specific metabolic functions. Among the most extensively studied candidates, *Megasphaera elsdenii* is a major lactate-utilizing bacterium capable of converting lactate into propionate, butyrate, and other short-chain fatty acids [96,97]. Because lactate accumulation is a key feature of ARA and may also occur transiently during adaptation to high-concentrate diets, supplementation with *M. elsdenii* has been investigated as a strategy to accelerate lactate clearance and stabilize ruminal fermentation. Aikman et al. [96] evaluated *M. elsdenii* NCIMB 41125 as a probiotic supplement in early-lactation dairy cows and demonstrated changes in ruminal pH and fermentation characteristics, supporting its potential role in modulating ruminal fermentation during dietary adaptation. However, the response to *M. elsdenii* may vary with strain, dose, timing of administration, diet composition, and the severity of the acidotic challenge. Other bacterial candidates may contribute through complementary metabolic functions. *Selenomonas ruminantium* is an important lactate-utilizing organism, but its lactate metabolism is strongly influenced by environmental pH and differs among strains. Fan et al. [97] demonstrated that lactate degradation by *S. ruminantium* and *M. elsdenii* was markedly affected by ambient pH under conditions relevant to SARA. In addition, Luo et al. [98], using seven strains of dairy propionibacteria, evaluated their potential to limit ruminal lactate accumulation and showed that their effects were strain dependent and were largely demonstrated under in vitro conditions. These findings indicate that bacterial probiotics should be selected according to demonstrated metabolic function and strain characteristics rather than taxonomic identity alone.

Yeast products represent a distinct class of microbial additives and should be differentiated from bacterial probiotics. Saccharomyces cerevisiae may improve ruminal conditions by supporting lactate-utilizing and fibrolytic populations and stabilizing fermentation under high-concentrate feeding. Nisbet and Martin [99] demonstrated that a S. cerevisiae culture stimulated lactate utilization by *S. ruminantium*, illustrating an indirect mechanism by which yeast may support resident lactate-utilizing bacteria. However, responses vary among live yeast cultures, fermentation products, cell-wall preparations, and other yeast-derived products because these preparations differ substantially in composition and biological activity. Postbiotics provide a further alternative to live microbial supplementation. They comprise non-viable microbial cells, cell components, fermentation products, enzymes, and other bioactive compounds that can influence microbial interactions and host responses without requiring persistent colonization [100]. Guo et al. [14] reported that yeast-derived postbiotics stabilized the solids-associated rumen microbiota and promoted microbial network interactions during grain-based SARA challenges. Overall, bacterial probiotics, yeast products, and postbiotics act through partly distinct mechanisms. Bacterial probiotics primarily aim to restore specific metabolic functions such as lactate utilization, whereas yeast products mainly modify the ruminal environment and support resident microbial populations, and postbiotics act through microbial- or host-active components. These intervention categories should therefore be evaluated separately rather than treated as functionally equivalent microbial additives.

### 6.4. Plant-Derived Bioactive Compounds and Functional Additives

Plant-derived compounds may influence ruminal acidosis through both microbial and host-directed mechanisms. Tannins, essential oils, saponins, flavonoids, and polyphenols can alter microbial growth, fermentation pathways, oxidative stress, and inflammatory responses [101,102]. Tannins can bind proteins and microbial enzymes and may reduce excessively rapid fermentation at appropriate doses [101]. However, excessive tannin intake can suppress beneficial microbial activity, decrease feed intake, and impair nutrient digestibility. Essential-oil components such as thymol, eugenol, cinnamaldehyde, and carvacrol possess antimicrobial properties and may selectively modify starch fermentation or VFA profiles [102]. Their effects are highly dose-dependent, and ruminal microorganisms may adapt during prolonged exposure, reducing the initial response. Flavonoids and polyphenols may provide additional antioxidant and anti-inflammatory effects and potentially protect epithelial integrity during acidotic stress [103]. Nevertheless, evidence linking these host effects directly to reduced ruminal acidosis remains less consistent than evidence for changes in oxidative or inflammatory markers.

### 6.5. Precision Microbiome Modulation

Precision microbiome modulation seeks to restore specific ecological functions rather than broadly suppress ruminal fermentation. Candidate approaches include rumen microbiota transplantation, defined microbial consortia, phage-based manipulation, selective enrichment of functional guilds, and microbiome-guided supplementation. These strategies may enable more targeted enhancement of lactate clearance, fiber degradation, hydrogen transfer, and ecosystem recovery than conventional nonspecific additive [31,104,105].

Rumen microbiota transplantation transfers complex microbial communities and their metabolic interactions, but successful engraftment and functional outcomes are strongly influenced by donor–recipient compatibility and host-specific ecological constraints. Experimental transfaunation studies have demonstrated substantial changes in microbial community structure, although fermentation and performance responses are variable and transplanted communities may gradually revert toward recipient-associated configurations [106]. Defined microbial consortia offer greater compositional and functional control by combining strains with complementary metabolic capacities. Recent evidence indicates that engineered ruminal consortia can enhance fiber degradation, but their performance depends on strain compatibility, metabolic cooperation, inoculum structure, and persistence within the resident community [105]. Phages and phage-derived enzymes may selectively suppress undesirable microbial populations. For example, the endolysin LyJH307 selectively inhibited *Streptococcus bovis* and altered carbohydrate-related fermentation pathways in vitro [107]. Nevertheless, phage-based interventions remain constrained by narrow host ranges, phage resistance, delivery limitations, and possible indirect effects on microbial networks [108]. Overall, precision microbiome modulation remains largely experimental and requires reproducible functional benefits, durable ecological effects, scalable production, rigorous biosafety assessment, and regulatory acceptance. The major mitigation strategies, together with their primary actions and key limitations, are compared in Table 3.

## 7. Future Perspectives

Future progress in ruminal acidosis research will require a transition from descriptive associations toward predictive, mechanistically validated, and practically applicable frameworks. Although ruminal pH will remain a central diagnostic variable, future systems should incorporate the magnitude, duration, and recovery trajectory of acidification and integrate these dynamics with feeding behavior, rumination, production performance, fermentation characteristics, microbial functions, and host response [1,17,72]. Candidate biomarkers should be classified according to whether they indicate pre-existing susceptibility, active ruminal dysfunction, or downstream epithelial injury and systemic inflammation, and their predictive value must be validated longitudinally under commercial production conditions [5,69]. At the mechanistic level, reliance on relative taxonomic abundance should be replaced by approaches combining absolute microbial quantification, metagenomics, metatranscriptomics, metaproteomics, targeted metabolomics, stable-isotope tracing, and defined-community experiments to distinguish microbial drivers of acidosis from compensatory responses or passive ecological markers [13,115]. Greater temporal resolution across baseline, dietary transition, established acidosis, and recovery phases will also be essential for separating causes from consequences and identifying the microbial functions responsible for ecosystem resistance and resilience [31]. Because animals exposed to similar diets differ substantially in pH dynamics, microbial adaptation, epithelial transport, inflammatory responses, and production losses, repeated measurements should be used to characterize stable susceptibility phenotypes and recovery capacity [20,114]. Ultimately, integrating continuous sensor data with functional microbial, metabolic, and host indicators may support interpretable prediction models and precision adjustment of fermentable carbohydrate supply, physically effective fiber, feeding frequency, buffers, and microbial interventions [17,60,72]. However, microbiome-based tools should demonstrate predictive value beyond existing farm data, remain robust across diets and production stages, and rely where possible on accessible matrices such as milk, blood, saliva, or feces [1,28]. Standardized definitions, multicenter validation, cross-herd testing, and economically feasible interventions will therefore be critical for detecting declining microbial and epithelial resilience before substantial health and production losses occur.

## 8. Conclusions

Ruminal acidosis is a multifactorial disorder driven by interactions among dietary fermentability, microbial metabolism, buffering, epithelial absorption, and host adaptation. ARA is typically associated with rapid lactate accumulation and severe acidification, whereas SARA more often involves recurrent VFA-driven pH depression and persistent subclinical effects. High-concentrate feeding disrupts microbial functional guilds, weakens metabolic cross-feeding, suppresses fibrolytic activity, and reduces ecosystem resilience. These changes alter organic acid metabolism and increase exposure to LPS, histamine, and other bioactive products, thereby promoting epithelial barrier dysfunction, inflammatory activation, hepatic stress, and production losses. Prevention and early intervention should therefore receive greater emphasis than short-term correction of ruminal pH alone. Gradual dietary adaptation, adequate physically effective fiber, appropriate carbohydrate management, and stage-specific feeding strategies remain the foundation of prevention. Microbiota-targeted approaches, including bacterial probiotics, yeast products, postbiotics, and other functional additives, may provide complementary benefits by improving lactate utilization, supporting fibrolytic activity, and enhancing microbial ecosystem stability. These interventions should complement, rather than replace, sound dietary management. Future strategies should integrate production-stage-specific nutrition, microbiota modulation, continuous ruminal monitoring, and host phenotyping to enable earlier risk detection and more precise prevention of ruminal acidosis.

## Figures and Tables

**Table 1:** Major microbial functional groups and ecological alterations associated with ruminal acidosis.

Microbial Functional Group	Representative Taxa	Common Response During Acidosis	Main Ecological Implication	References
Starch-degrading and lactate-producing bacteria	*Streptococcus bovis*, *Lactobacillus* spp.	Generally increased, particularly during rapid carbohydrate overload	Associated with accelerated starch fermentation and lactate production and may contribute to severe acidification in ARA	[9,10,11,37]
Lactate-utilizing bacteria	*Megasphaera elsdenii*, *Selenomonas ruminantium*	Variable or delayed response	Convert lactate into propionate and other VFAs; delayed activity may contribute to insufficient lactate clearance, particularly during ARA or abrupt adaptation to highly fermentable diets	[10,38,40]
Fibrolytic bacteria	*Fibrobacter succinogenes*, *Ruminococcus albus*, *R. flavefaciens*	Generally decreased in abundance or activity	Reduced cellulose degradation, weakened fiber-associated interactions, and lower fermentation stability	[9,41,42]
Metabolically versatile bacteria	*Prevotella* spp.	Inconsistent among studies	Responses depend on species-level differences in starch, protein, peptide, and hemicellulose utilization	[6,36,54]
Rumen protozoa	*Entodinium*, *Diplodinium and related taxa*	Frequently decreased, but variable	Altered starch sequestration, bacterial predation, and hydrogen metabolism	[46]
Anaerobic fungi	*Neocallimastigomycota members*	Activity generally reduced under sustained low pH	Impaired initial colonization and degradation of fibrous plant tissues	[55]
Methanogenic archaea	*Methanobrevibacter and related methanogens*	Methanogenic activity often reduced	Disturbed interspecies hydrogen transfer and altered fermentation redox balance	[12]

Note: Reported responses vary with animal species, dietary substrate, acidosis model, sampling time, ruminal fraction, and analytical method. Changes in relative abundance do not necessarily represent changes in absolute abundance or metabolic activity. ARA, acute ruminal acidosis; VFA, volatile fatty acid.

**Table 2:** Major fermentation metabolites and microbial-derived bioactive products associated with ruminal acidosis.

Metabolite or Product	Common Response During Acidosis	Main Biological Relevance	References
Total VFAs	Frequently increased during SARA	Increase ruminal acid load and contribute to recurrent pH depression	[1,6,16,58,60]
Acetate/propionate ratio	Generally decreased during high-concentrate feeding	Reflects a shift from fibrolytic toward starch-associated fermentation	[16,38,69]
Lactate	Markedly increased in ARA but generally transient or low in SARA	Rapid accumulation causes severe acidification and impairs microbial and epithelial functions	[9,10,11,37,39]
Succinate and related intermediates	May accumulate when microbial cross-feeding is disrupted	Indicate incomplete metabolite transfer and altered propionate formation	[6,40]
Lipopolysaccharide	Frequently increased, particularly in grain-induced models	Activates innate immune signaling and may impair epithelial barrier integrity	[28,29,59,60,64]
Histamine and other biogenic amines	Frequently increased during rapid carbohydrate or amino acid fermentation	May promote epithelial injury, inflammation, and vascular dysfunction	[62,64,70]
Tryptophan-derived metabolites	Altered profiles have been reported	May regulate epithelial signaling, immunity, and microbial host communication	[67,68]
Biohydrogenation intermediates	Altered during high-concentrate feeding	Certain intermediates suppress mammary lipid synthesis and contribute to milk fat depression	[69,71]

Note: ARA, acute ruminal acidosis; SARA, subacute ruminal acidosis; VFA, volatile fatty acid.

**Table 3:** Major strategies for preventing or mitigating ruminal acidosis.

Strategy	Main Action	Key Limitation	References
Dietary adaptation and physically effective fiber	Regulate fermentable substrate supply and enhance rumination, salivary buffering, and microbial adaptation	Effectiveness depends on diet formulation, feed processing, and feeding behavior	[9,18,58,88,89]
Buffers and alkalizing agents	Neutralize ruminal acids and reduce the duration of low-pH exposure	Do not correct the underlying dietary imbalance	[55,93,94,95]
Probiotics, directly fed microbials, and yeast products	Promote lactate utilization, fibrolytic activity, and fermentation stability	Effects are strain, product, diet, and host-dependent	[38,40,55,109,110]
Postbiotics	Modulate microbial interactions and host responses through stable bioactive components	Product composition, active components, mechanisms of action, and optimal doses remain insufficiently defined	[14,111]
Plant-derived bioactive compounds	Modulate microbial fermentation, oxidative stress, and inflammatory responses	Strong dose dependence and variable product composition	[101,102,103]
Precision microbiome modulation	Restore or selectively manipulate microbial functions using transplantation, defined consortia, or phages	Limited validation, uncertain persistence, and biosafety concerns	[112,113,114]

Note: The efficacy of these strategies varies with diet composition, animal susceptibility, dosage, treatment duration, and experimental conditions.

## Data Availability

No new data were created or analyzed in this study. Data sharing is not applicable to this article.

## Abbreviations

The following abbreviations are used in this manuscript:

ARA	Acute ruminal acidosis
SARA	Subacute ruminal acidosis
VFA	Volatile fatty acid
LPS	Lipopolysaccharide
NDF	Neutral detergent fiber
TLR4	Toll-like receptor 4
MD-2	Myeloid differentiation factor 2
NF-κB	Nuclear factor kappa B
MAPK	Mitogen-activated protein kinase
TNF-α	Tumor necrosis factor alpha
IL-1β	Interleukin-1 beta
IL-6	Interleukin-6
